# The role of staging laparoscopy before therapy in patients with pancreatic ductal adenocarcinoma: a real-world, single-center experience

**DOI:** 10.3389/fmed.2026.1776139

**Published:** 2026-03-16

**Authors:** Koji Kikuchi, Akira Umemura, Hiroyuki Nitta, Daiki Takeda, Taro Ando, Satoshi Amano, Toma Kawashima, Taku Kimura, Taku Kodama, Akira Sasaki

**Affiliations:** Department of Surgery, Iwate Medical University, Morioka, Japan

**Keywords:** chemoradiotherapy, neoadjuvant chemotherapy, pancreatic ductal adenocarcinoma, resectability status, staging laparoscopy

## Abstract

**Background:**

Staging laparoscopy (SL) for pancreatic ductal adenocarcinoma (PDAC) is considered useful in improving the accuracy of staging and resectability. However, currently, there are no standard criteria for selecting patients who may benefit from SL prior to determining the appropriate treatment. In this report, we aimed to determine the role of SL before therapy in patients with PDAC.

**Methods:**

This study was a single-center, prospective cohort study comprising patients with PDAC at Iwate Medical University Hospital during the period from November 2021 to June 2025. SL was performed in all patients with PDAC with no radiological distant metastasis before they underwent pancreatic resection or chemotherapy or chemoradiotherapy. The baseline characteristics, operative outcomes, changes of resectability status, complete resection rate and mid-term survival were examined.

**Results:**

102 patients were finally included in the present study. Before SL, according to resectability status, 45 patients (44.1%) were classified as resectable (R), 36 (35.0%) as borderline resectable (BR), and 21 (20.6%) as unresectable locally advanced (UR-LA). SL revealed distant metastasis in 24 (23.5%) patients. The univariate analysis revealed that the factors of CEA positive and CA19-9 ≥ 150, U/mL were associated with a significantly higher risk of occult metastasis. The multivariate analysis revealed that having a CEA positive and CA19-9 ≥ 150, U/mL were the only factors that were independently associated with occult metastasis. For patients with R PDAC before SL, distant metastasis was found after SL in 17.8% of patients. Even in cases where none of the tumor markers were elevated, one case was found to have distant metastasis after SL. There was no morbidity and mortality in this case series. In the intention-to-treat population, the median overall survival (OS) were not reached [95% CI: 22.0 months–not estimable (NE)]. The median DFS was 22.0 months (95% CI: 15.0 months–27.0 months). Of the patients with UR-LA PDAC, despite stage migration, no improvement in OS was observed after SL.

**Conclusion:**

SL is safe and effective in determining accurate staging, which may allow for more appropriate treatment. Therefore, SL is actively recommended for patients with R PDAC or BR PDAC who are planning to undergo complete resection shortly to avoid unnecessary surgical exploration, especially those CEA positive or with CA19-9 ≥ 150 U/mL.

## Introduction

Pancreatic ductal adenocarcinoma (PDAC) has one of the worst prognoses of all cancers. According to the National Comprehensive Cancer Network (NCCN) Clinical Practice Guidelines, PDAC is categorized into upfront resectable, borderline resectable, locally advanced, or metastatic disease (1). Although curative surgical resection is the only option for achieving a cure, only approximately 15–20% of all patients with PDAC have upfront resectable or borderline resectable disease (2). Positive cytology from peritoneal washings obtained prior to the potential resection of PDAC are associated with a grim prognosis, equivalent to metastatic disease (3). Evaluation of cytology findings from peritoneal washings, which is important for preparing a treatment strategy, cannot be undertaken before staging laparoscopy (SL).

SL is a minimally invasive procedure used to assess macroscopic or occult metastases of abdominal tumors cancers including esophageal, gastric, pancreatic and periampullary, hepatic, and biliary tract cancers (4). By performing SL, unnecessary surgical exploration can be avoided, and systemic therapy can be rapidly introduced in patients with occult metastasis (5).

SL for PDAC is considered useful in improving the accuracy of staging and resectability. However, currently, no standard criteria have been established for selecting patients who may benefit from SL as part of pre-operative staging. In this study, we aimed to determine the role of SL before therapy in patients with PDAC.

## Materials and methods

### Data collection and study design

We conducted a single-center, prospective cohort study comprising patients with PDAC who attended Iwate Medical University Hospital between November 2021 and June 2025. Patients with PDAC who were under 80 years old, had no other malignant tumors, and had no distant metastasis were included in the study. The pathological diagnosis of PDAC relies on endoscopic ultrasonography-guided fine needle aspiration biopsy or endoscopic retrograde cholangiopancreatography. Patients who had undergone upfront surgery due to reasons such as difficulty administering chemotherapy or patient preference were exclude in the study. This is because SL was not performed on these patients, and the baseline characteristics was biased, making it difficult to compare them with the SL group. Preoperative resectability was evaluated using triple-phase helical computed tomography (CT), gadolinium ethoxy benzyl diethylenetriamine pentaacetic acid-enhanced magnetic resonance imaging, and fluorodeoxyglucose-positron emission tomography. According to resectability status, patients were classified as resectable (R), borderline resectable (BR), unresectable locally advanced (UR-LA), or unresectable metastasis (UR-M) (1). The definition of complete resection is R0 resection. Positive cytology was defined as a finding of malignant cells in peritoneal washings collected or atypical cells that were highly concerning for malignancy by independent review by our pathology department and multidisciplinary case conference consensus. Patients with positive cytology were classified as UR-M and patients with atypical cells that were not considered positive cytology were treated as negative cytology. We graded postoperative morbidity on the basis of the Clavien-Dindo classification, and grade ≥ IIIa events were counted as postoperative complications (6). This study protocol was performed in accordance with the Declaration of Helsinki. The ethics committee of Iwate Medical University Hospital approved the study (reference MH2019-123 and MH2020-129). Informed consent was obtained from all included patients.

### Therapeutic strategy

**FIGURE 1:**
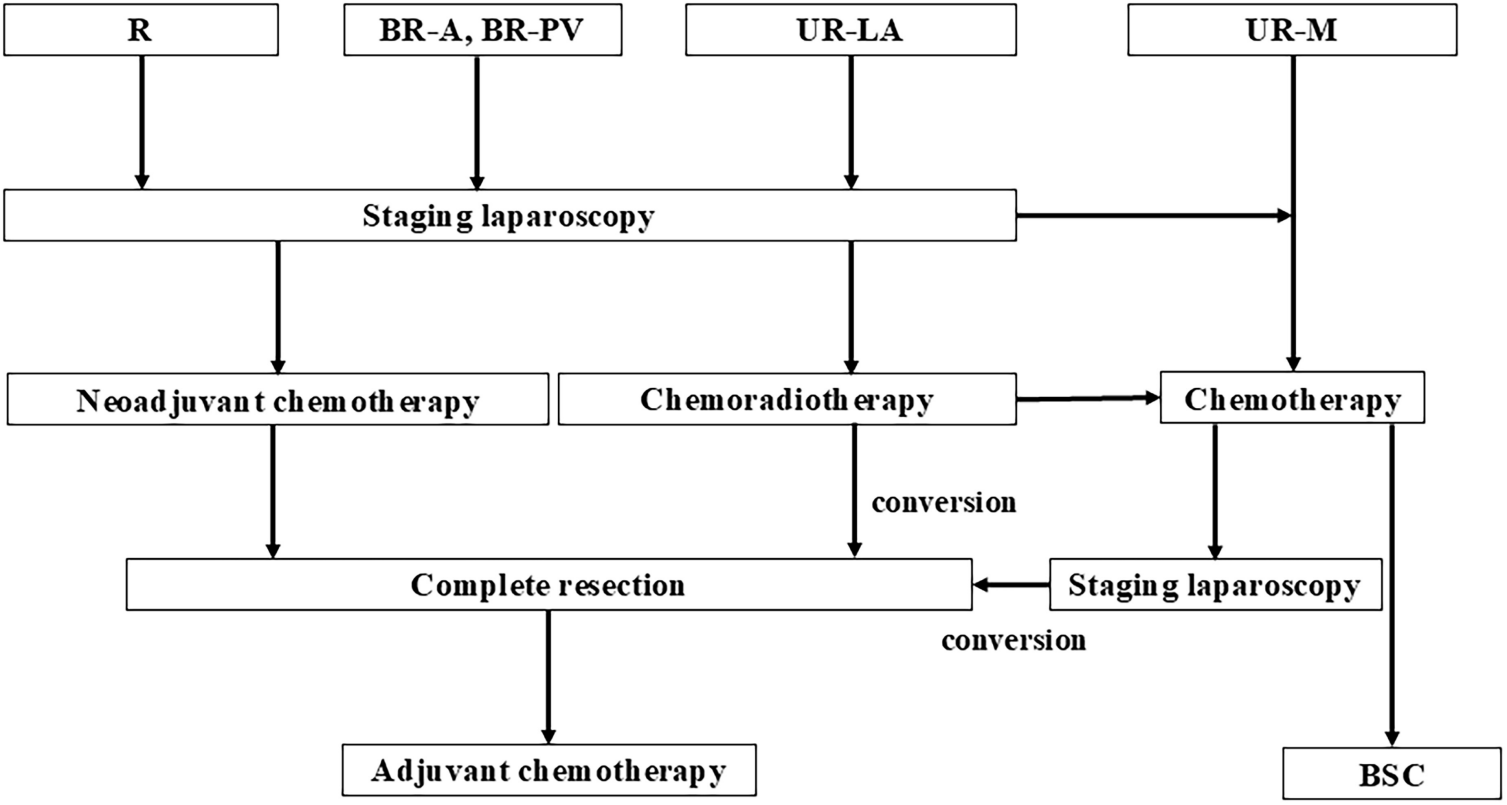
The flow chart of pancreatic ductal adenocarcinoma patients treated at Iwate Medical University Hospital. R, resectable; BR-A, borderline resectable arterial invasion; BR-PV, borderline resectable portal vein invasion; UR-LA, unresectable locally advanced; UR-M, unresectable metastasis; BSC, best supportive care.

SL was performed on all patients with PDAC who had no radiological distant metastasis before they underwent pancreatic resection or chemotherapy. In cases of R or BR status after SL, triple-phase helical CT evaluation was performed after two courses of nab-paclitaxel plus gemcitabine (GnP) therapy as neoadjuvant chemotherapy (NAC). A 30- min intravenous infusion of nab- paclitaxel (125 mg/m^2^) was administered to all enrolled patients, followed by a 30- min intravenous infusion of gemcitabine (1,000 mg/m^2^), on days 1, 8, and 15 over a 4-week period as one regimen cycle. In the absence of distant metastasis or progression to UR-LA status, complete resection was performed followed by postoperative adjuvant chemotherapy with S-1 monotherapy in all patients. The regimen consisted of S-1 80–120 mg/ day, depending on the body surface area (<1.25 m^2^: 80 mg/day, 1.25–1.50 m^2^: 100 mg/day, and > 1.50 m^2^: 120 mg/day), administered twice a day for 4 weeks followed by withdrawal for 2 weeks and repeated every 6 weeks for 4 courses, or administered twice a day for 2 weeks followed by withdrawal for 1 week and repeated every 3 weeks for 8 cycles. In the case of UR-LA diseases, if imaging evaluation after two courses of the GnP or FOLFIRINOX (oxaliplatin, 85 mg per square meter of body-surface area; irinotecan, 180 mg per square meter; leucovorin, 400 mg per square meter; and fluorouracil, 400 mg per square meter given as a bolus followed by 2,400 mg per square meter given as a 46-h continuous infusion, every 2 weeks) regimen and radiation therapy indicated that conversion surgery was possible, complete resection was performed within 6 weeks of the end of the systemic chemotherapy. Radiation therapy was administered in 1.8 Gy fractions, once a day, 5 days a week, for a total of 28 fractions (50.4 Gy). Our criteria for conversion surgery are as follows: patients showing adequate reduction of the main tumor, enabling complete removal inclusive of the major vessels, those with tumor markers normalized or at least several months of local control, those with no metastasis. If resection was deemed impossible after two courses of systemic chemotherapy and radiation therapy, systemic chemotherapy was continued as a general rule. If conversion surgery was deemed possible during the course of treatment, the transition to surgical treatment was considered. In the case of UR-M status, systemic chemotherapy was performed. The treatment policy was changed depending on the evaluation of the treatment effect, and the treatment strategy was set to aim for complete resection (Figure 1).

### Surgical procedures

**FIGURE 2:**
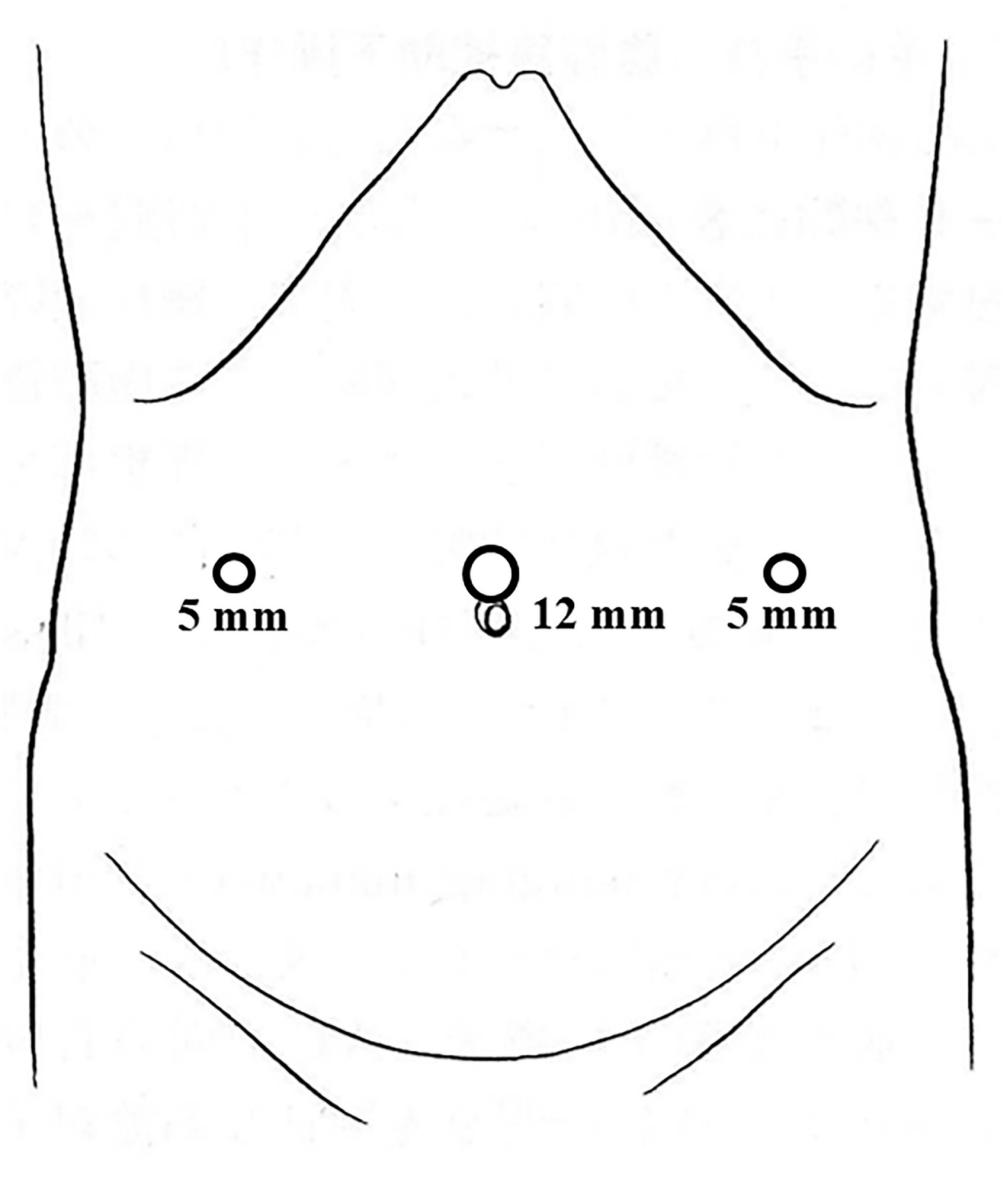
Port placement for staging laparoscopy.

The patient was placed in a supine position. The surgeon stood on the patient’s right side, while the scopist stood on the patient’s left side. A 12-mm port was placed in the umbilical position. Two 5-mm trocar ports were placed in the same transverse plane as the umbilical port, approximately 5–7 cm away (Figure 2).

**FIGURE 3:**
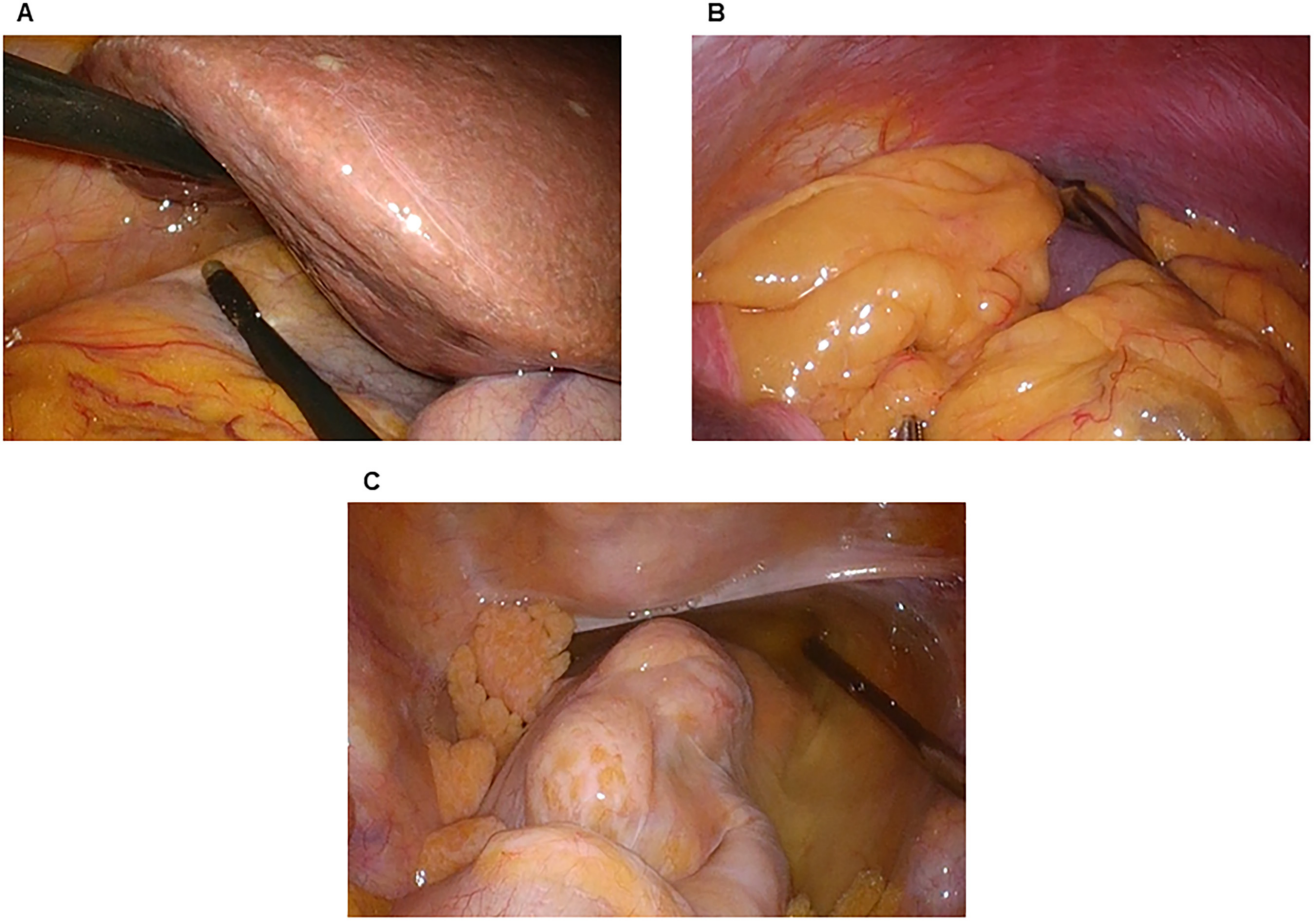
Peritoneal washing cytology. **(A)** The pouch of Morrison. **(B)** The left subdiaphragmatic area. **(C)** The pouch of Douglas.

**FIGURE 4:**
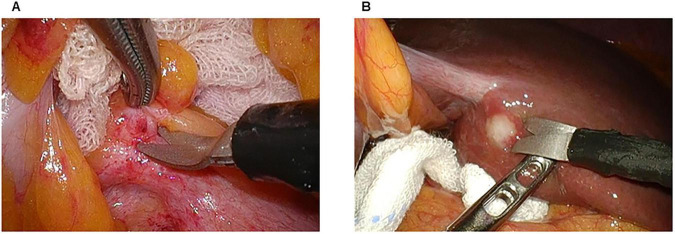
Biopsy of peritoneal dissemination and liver metastasis. **(A)** Peritoneal dissemination. **(B)** Liver metastasis.

First, we observed any disseminated nodules within the abdominal cavity, infiltration into the transverse mesocolon, and metastasis on the surface of the liver. Second, we performed a cytological examination of peritoneal washings with 50 mL of normal saline aspirated from the pouch of Morrison (Figure 3A), the left subdiaphragmatic area (Figure 3B), and the pouch of Douglas (Figure 3C). If metastasis or dissemination was found during surgery, a biopsy was performed (Figures 4A,B). In cases where dissemination or metastasis was found, additional procedures such as central venous catheter construction or gastrojejunal bypass were performed.

### Statistical analysis

Data are presented as mean ± standard deviation (SD), and categorical variables are described as totals and frequencies. Differences in the patient groups were assessed using the Mann–Whitney U test for continuous variables and the chi-square or Fisher exact test (for expected counts of < 5) for categorical variables. Overall survival (OS) was defined as the time from the initial treatment to death and expunged at the date of loss to follow-up or data cut-off. Disease-free survival (DFS) was defined as the time from the complete resection to recurrence and expunged at the date of loss to follow-up or data cut-off. Survival was estimated using the Kaplan–Meier method, and the survival difference was compared using the log-rank test. We performed a multivariate logistic regression analysis using variables with *p* < 0.05 in the univariate analysis. A statistical analysis was performed using the JMP version 14.2.0 software (SAS Institute, Cary, NC). Variables with *p* < 0.05 were considered statistically significant.

## Results

**FIGURE 5:**
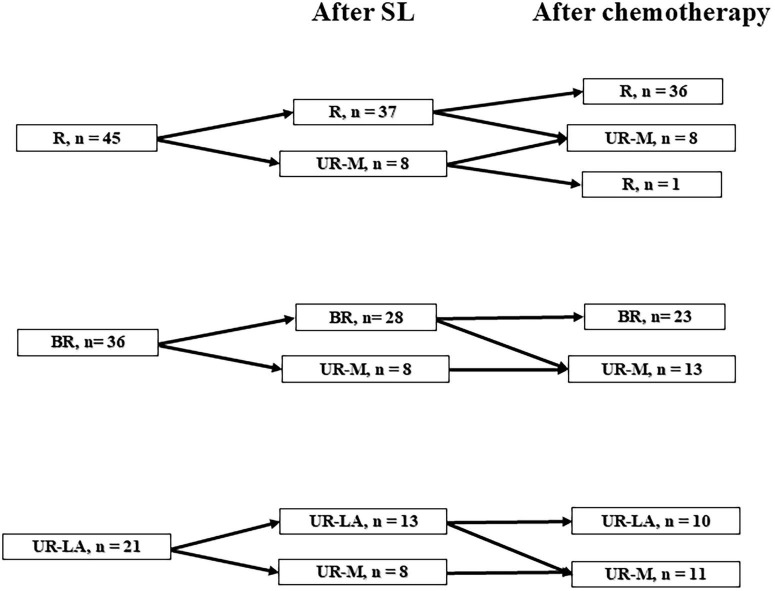
Tree diagram showing the change in resectability status. R, resectable; BR, borderline resectable; UR-LA, unresectable locally advanced; UR-M, unresectable metastasis.

The baseline characteristics and perioperative outcomes are described in Table 1. Of the total of 102 patients included in the study, 59 were male and 43 were female. The patient’s mean age was 66.6 ± 8.8 years. In 77 patients (75.5%), the tumor was located in the pancreatic head. Before SL, according to resectability status, 45 patients (44.1%) were classified as R, 31 (30.1%) as BR portal vein invasion (BR-PV), 5 (4.9%) as BR arterial invasion (BR-A), and 21 (20.6%) as unresectable locally advanced (UR-LA); (1). The mean operative time was 70.2 ± 42.8 min, and the mean intraoperative blood loss was 4.7 ± 14.5 mL. SL revealed distant metastasis in 24 patients (23.5%). In the evaluation after chemotherapy, 9 patients bacame UR-M, while 1 patient of UR-M became R. Figure 5 shows a tree diagram of the change in resectability status. The mean number of days from SL to the start of chemotherapy was 14.8 ± 8.5 days. No morbidity or mortality was found in this case series (Table 1).

**TABLE 1:** Characteristics and operative outcomes.

Variables	*n* = 102
Age (years)	66.6 ± 8.8
Sex (Male/Female)	59/43
BMI (kg/m^2^)	20.7 ± 4.0
**ASA (n, %)**	
I	7 (6.9%)
II	87 (85.3%)
III	8 (7.8%)
**Location of tumor (n, %)**	
Ph	77 (75.5%)
Pb	21 (20.6%)
Pt	4 (3.9%)
**Resectability status before SL (n, %)**	
R	45 (44.1%)
BR-PV	31 (30.1%)
BR-A	5 (4.9%)
UR-LA	21 (20.6%)
CEA (ng/mL)	8.3 ± 43.4
CA19-9 (U/mL)	674.1 ± 2689.
SPan-1 (U/mL)	219.1 ± 406.5
DUPAN-2 (U/mL)	429.6 ± 700.2
Operative time (min)	70.2 ± 42.8
Blood loss (mL)	4.7 ± 14.5
Morbidity (Clavien-Dindo ≥ grade III) (n, %)	0 (0%)
Atypical cell from peritoneal washings (n, %)	18 (17.6%)
Metastasis detection (n, %)	24 (23.5%)
Liver metastasis	12 (11.8%)
Peritoneal malignancy	5 (4.9%)
Positive cytology from peritoneal washings	14 (13.7%)
**Change of resectability status after SL (n, %)**	
R → UR-M	8 (7.8%)
BR-A, BR-PV → UR-M	8 (7.8%)
UR-LA → UR-M	8 (7.8%)
NC	78 (76.5%)
**Change of resectability status after chemotherapy (n, %)**	
R → UR-M	1 (1.0%)
BR-A, BR-PV → UR-M	5 (4.9%)
UR-LA → UR-M	3 (2.9%)
UR-M → R	1 (1.0%)
NC	92 (90.2%)
Duration until chemotherapy begins (days)	14.8 ± 8.5
**Chemotherapy regimen (n, %)**	
GnP	71 (78.9%)
FOLFIRINOX	15 (16.7%)
GS	4 (4.4%)
Complete resection performed (n, %)	62 (60.8%)
Mortality (n, %)	0 (0%)

Values are expressed as means ± SDs. BMI, body mass index; ASA, American Society of Anesthesiologists;Ph, pancreatic head; Pb, pancreatic body; Pt, pancreatic tail; SL, staging laparoscopy; R, resectable; BR-PV, borderline resectable portal vein invasion; BR-A, borderline resectable arterial invasion; UR-LA, unresectable locally advanced; CEA, carcinoembryonic antigen; CA19-9, carbohydrate antigen 19–9; UR-M, unresectable metastasis; NC, no change; GnP, nab-paclitaxel plus gemcitabine; GS, S-1 plus gemcitabine.

The univariate analysis revealed that the factors of CEA positive and CA19-9 ≥ 150, U/mL were associated with a significantly higher risk of occult metastasis. The multivariate analysis revealed that having a CEA positive and CA19-9 ≥ 150, U/mL were the only factors that were independently associated with occult metastasis (Table 2). Of the patients with R PDAC before SL, 17.8% were found distant metastasis after SL. Even among the cases without the tumor marker, one was found to have distant metastasis after SL (Table 3).

**TABLE 2:** Univariate and multivariate analysis (predictors of occult metastasis).

Variables, (%)	Univariate analysis			Multivariate analysis		
	No change (*n* = 78)	UR-M after SL (*n* = 24)	*P*	Odds ratio	95 % CI	*P*
Age **≥** 65, years	47 (60.3%)	16 (66.7%)	0.570			
Male	44 (56.4%)	15 (62.5%)	0.596			
BMI **≥** 23, kg/m^2^	20 (25.6%)	7 (29.2%)	0.734			
Tumor size **≥** 3, cm	23 (29.5%)	11 (45.8%)	0.144			
Location of tumor			0.332			
Ph	58 (74.4%)	19 (79.2%)				
Pb	16 (20.5%)	5 (20.8%)				
Pt	4 (5.1%)	0 (0.0%)				
Resectability status before SL (n, %)			0.210			
R	37 (47.4%)	8 (33.3%)				
BR	28 (35.9%)					
UR-LA	8 (33.3%)	13 (16.7%)	8 (33.3%)			
Stage before SL (n, %)			0.224			
I	23 (29.5%)	4 (16.7%)				
II	33 (42.3%)	9 (37.5%)				
III	22 (28.2%)	11 (45.8%)				
CEA positive (n, %)	12 (15.4%)	10 (43.5%)	0.007	3.334	1.145–9.706	0.027
CA19-9 positive (n, %)	60 (76.9%)	18 (75.0%)	0.847			
SPan-1 positive (n, %)	55 (71.4%)	19 (86.4%)	0.135			
DUPAN-2 positive (n, %)	31 (40.3%)	12 (54.6%)	0.235			
CA19-9 ≥ 150, U/mL	30 (38.5%)	17 (70.8%)	0.005	2.952	1.048-8.313	0.040

UR-M, unresectable metastasis; SL, staging laparoscopy; CI, confidence interval; BMI, body mass index; Ph, pancreatic head; Pb, pancreatic body; Pt, pancreatic tail; R, resectable; BR, borderline resectable; UR-LA, unresectable locally advanced; CEA, carcinoembryonic antigen; CA19-9, carbohydrate antigen 19–9.

**TABLE 3:** Comparison of tumor markers in patients with R pancreatic cancer before staging laparoscopy.

Variables	R after SL (*n* = 37)	UR-M after SL (*n* = 8)	*P*
CEA (ng/mL)	3.2 ± 4.4	4.2 ± 2.1	0.038
CA19–9 (U/mL)	158.1 ± 184.5	715.8 ± 900.4	0.172
SPan-1 (U/mL)	202.4 ± 404.8	236.6 ± 303.1	0.211
DUPAN-2 (U/mL)	115.7 ± 164.2	544.4 ± 727.9	0.143
CEA positive (n, %)	2 (5.4%)	3 (37.5%)	0.022
CA19-9 positive (n, %)	25 (67.6%)	6 (75.0%)	0.676
SPan-1 positive (n, %)	27 (79.4%)	7 (87.5%)	0.306
DUPAN-2 positive (n, %)	9 (24.3%)	4 (50.0%)	0.162
At least one marker positive (n, %)	29 (78.4%)	7 (87.5%)	0.541

Values are expressed as means ± SDs. R, resectable; UR-M, unresectable metastasis; SL, staging laparoscopy; CEA, carcinoembryonic antigen; CA19-9, carbohydrate antigen 19–9.

In the resectability status evaluation before SL, the complete resection rate was 80.0% for the patients with R PDAC, 58.3% for those with BR-A and BR-PV PDAC, and 23.8% for the those with UR-LA PDAC. After SL, the complete resection rates were 94.6% for the patients with R PDAC, 75.0% for those with BR-A and BR-PV PDAC, 38.5% for those with UR-LA PDAC, and 4.2% for those with UR-M PDAC. After chemotherapy, the complete resection rates were 97.3% for the patients with R PDAC, 91.3% for those with BR-A and BR-PV PDAC, 62.5% for those with UR-LA PDAC, and 0% for patients with UR-M PDAC (Table 4).

**TABLE 4:** Comparison of complete resection rate between resectability status.

Variables	Complete resection rate
**Resectability status before SL (%, complete resection/n)**	
R	80.0% (36/45)
BR-A, BR-PV	58.3% (21/36)
UR-LA	23.8% (5/21)
**Resectability status after SL (%, complete resection/n)**	
R BR-A, BR-PV UR-LA UR-M	94.6% (35/37) 75.0% (21/28) 38.5% (5/13) 4.2% (1/24)
**Resectability status after chemotherapy (%, complete resection/n)**	
R	97.3% (36/37)
BR-A, BR-PV	91.3% (21/23)
UR-LA	62.5% (5/8)
UR-M	0.0% (0/34)

SL, staging laparoscopy; R, resectable; BR-A, borderline resectable arterial invasion; BR-PV, borderline resectable portal vein invasion; UR-LA, unresectable locally advanced; UR-M, unresectable metastasis.

**FIGURE 6:**
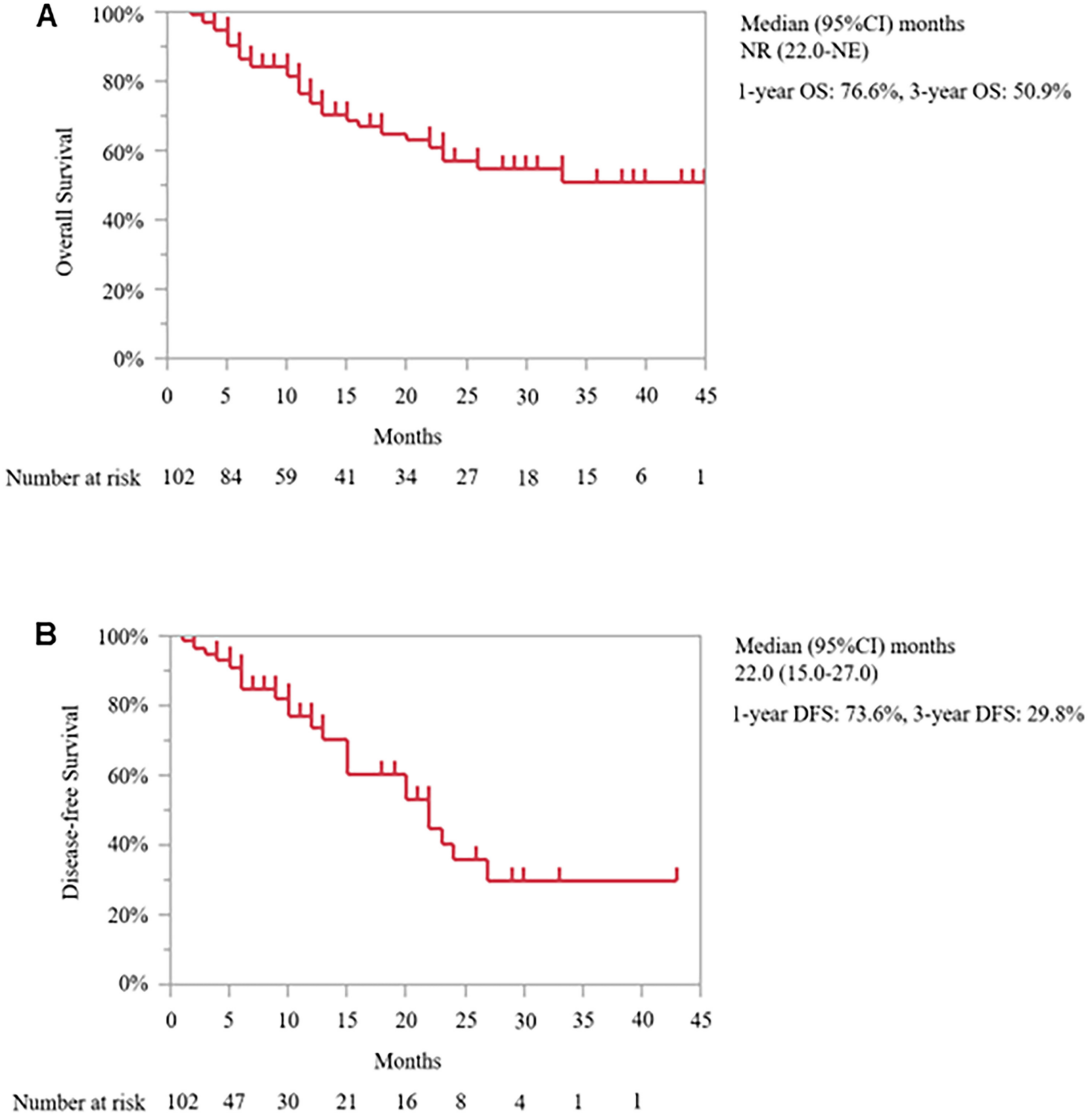
The Kaplan–Meier curve of overall survival **(A)** of the intention-to-treatment population in all patients and disease-free survival **(B)** of the intention-to-treatment population in patients who underwent complete resection. NR, not reached; NE, not estimable; OS, overall survival; DFS, disease-free survival

**FIGURE 7:**
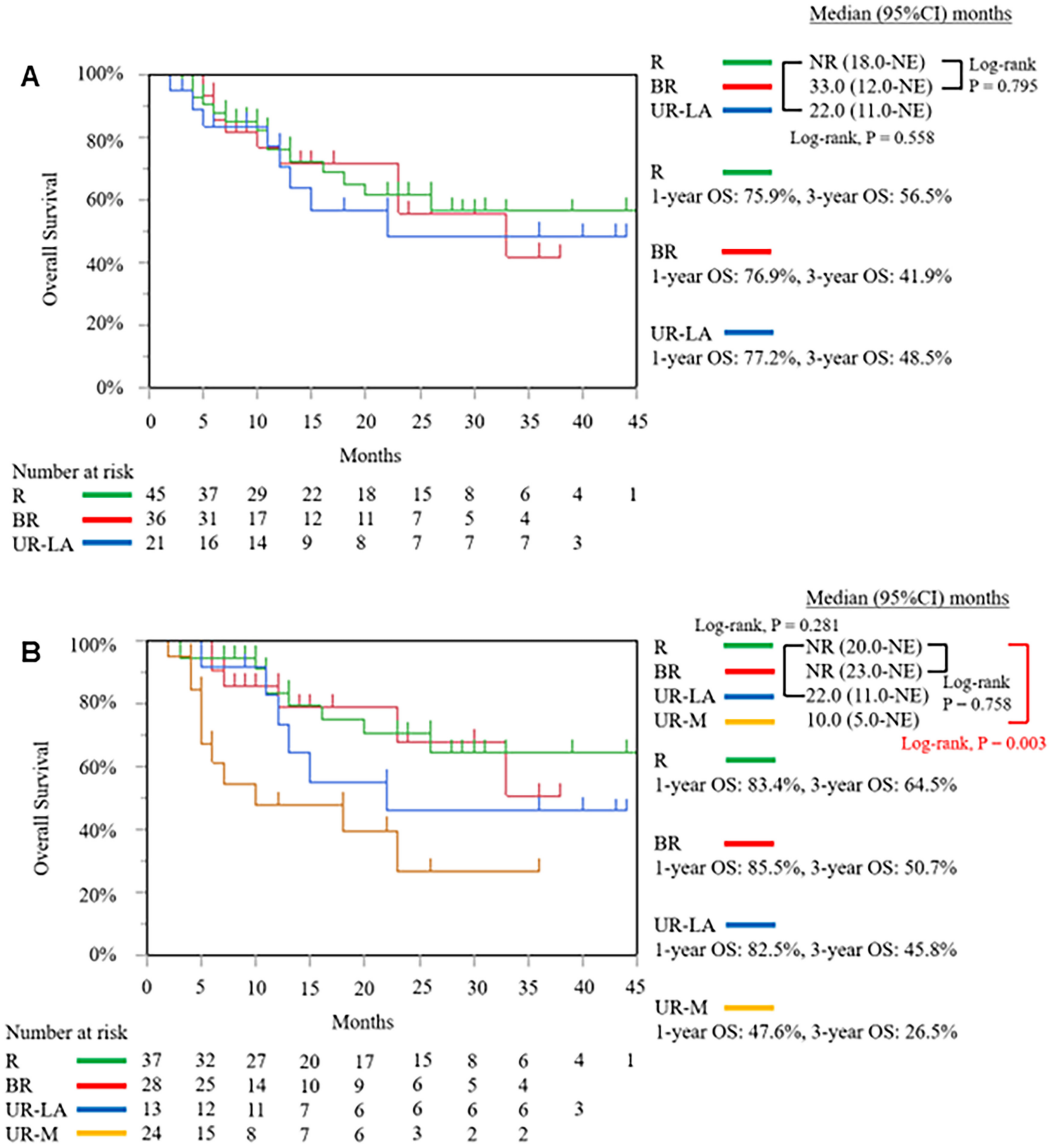
The Kaplan–Meier curve of overall survival of the intention-to-treatment population based on the resectability status before **(A)** and after staging laparoscopy **(B)**. NR, not reached; NE, not estimable; OS, overall survival; R, resectable; BR, borderline resectable; UR-LA, unresectable locally advanced; UR-M, unresectable metastasis

**FIGURE 8:**
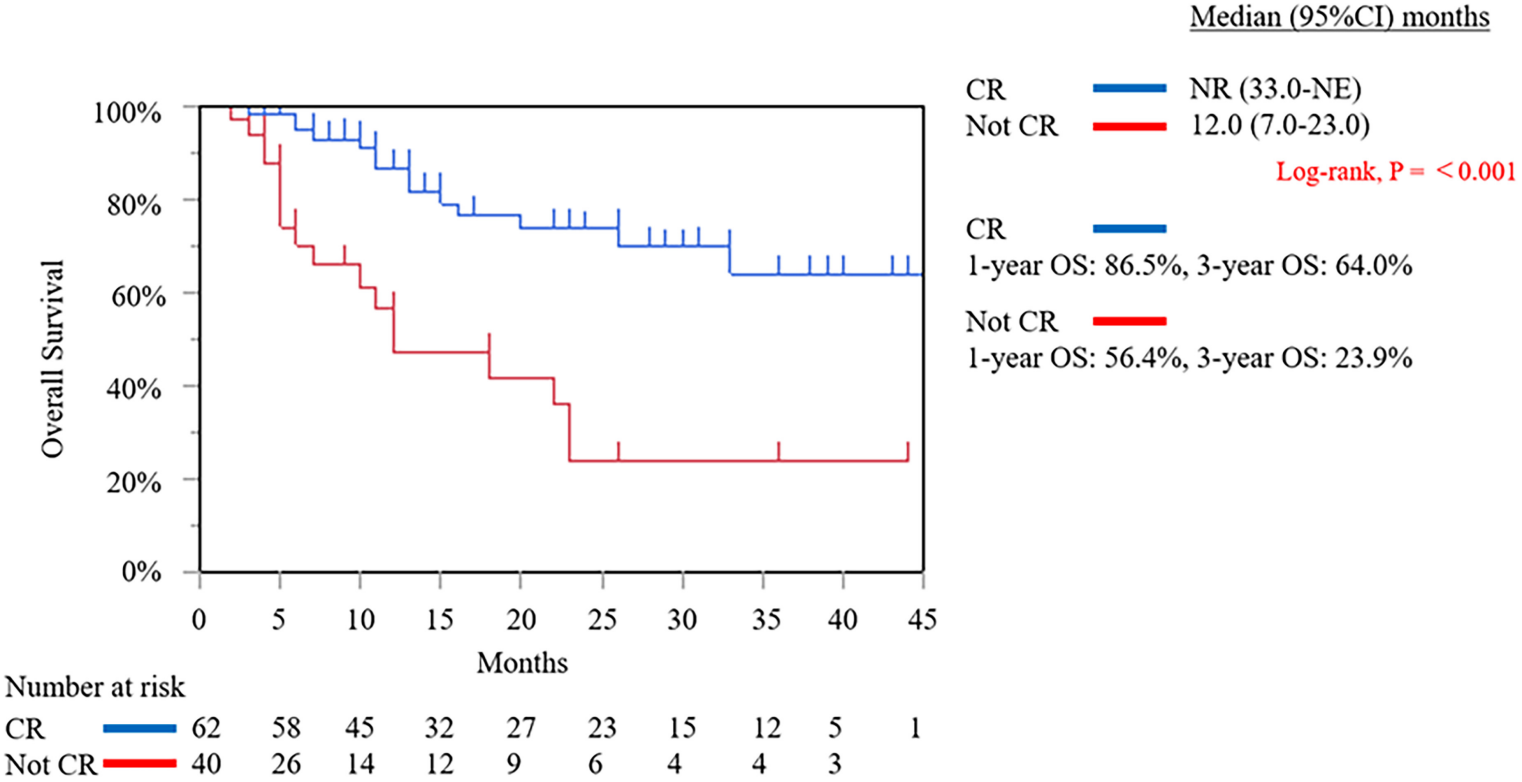
The Kaplan–Meier curve of overall survival of the intention-to-treatment population in complete resection group and not complete resection group. NR, not reached; NE, not estimable; OS, overall survival; CR, complete resection.

**FIGURE 9:**
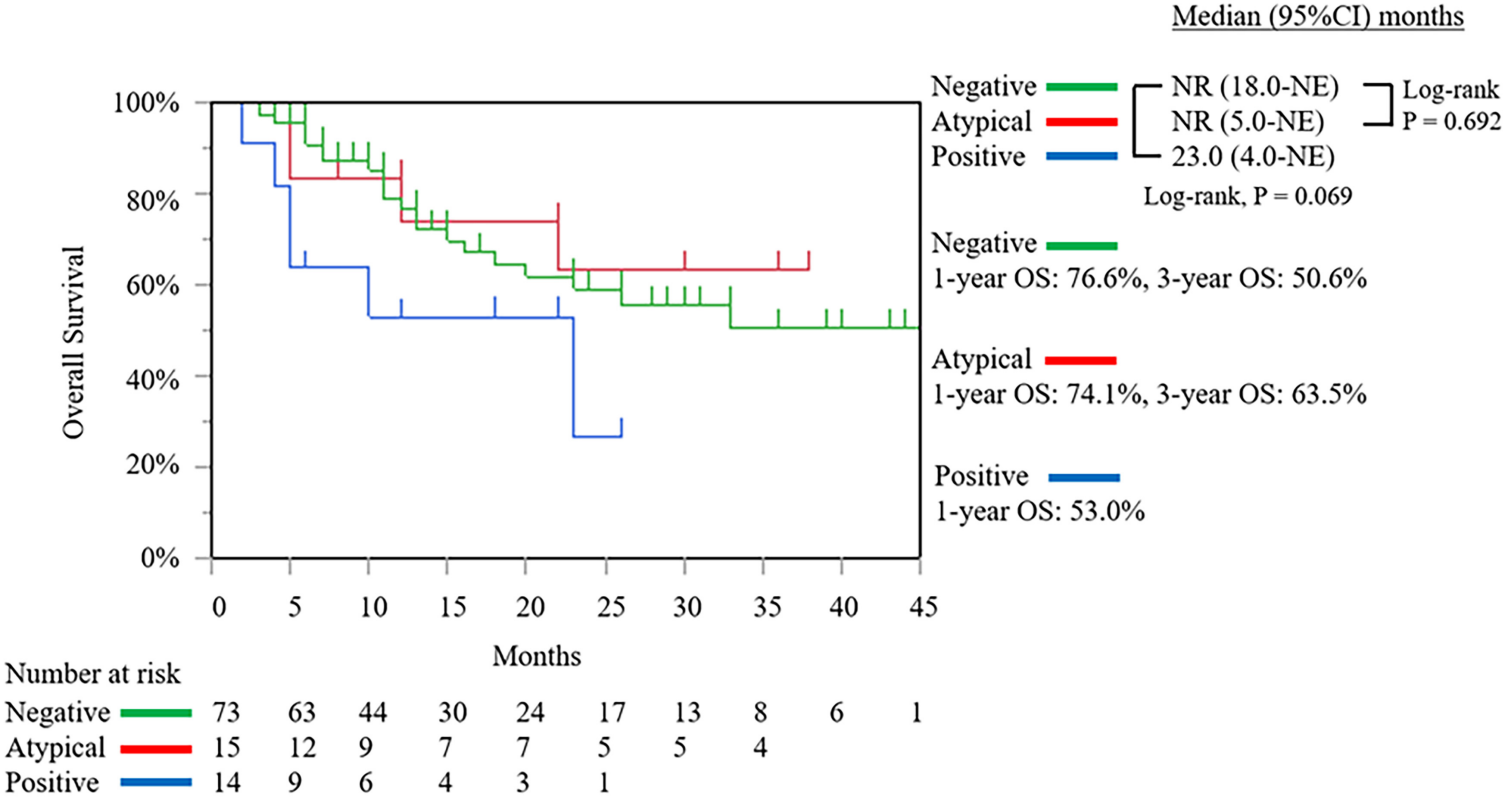
The Kaplan–Meier curve of overall survival of the intention-to-treatment population between the three groups according to the results of cytological examination of peritoneal washings. NR, not reached; NE, not estimable; OS, overall survival; Negative, negative cytology group; Atypical, atypical cells detected group; Positive, positive cytology group.

As of the data cut-off date of September 13, 2025 from November 9, 2021, the median follow-up duration was 11.0 months (interquartile range: 5.0–24.0 months). In the intention-to-treat population, the median OS was not reached [95% confidence interval (CI): 22.0 months–not estimable (NE)]. The 1-, and 3-year OS rates were 76.6, and 50.9%, respectively (Figure 6A). The median DFS was 22.0 months (95% CI: 15.0–27.0 months) (Figure 6B). We compared the outcomes based on the resectability status before and after SL. Before SL, the median OS of the R PDAC group was not reached (95% CI: 18.0 months–NE), which was not significantly different from the results obtained from the BR PDAC group (median OS 33.0 months; 95% CI: 12.0 months–NE; *P* = 0.795) and was UR-LA PDAC group (median OS 22.0 months; 95% CI: 11.0 months–NE; *P* = 0.558) (Figure 7A). After SL, the median OS of the R PDAC group was not reached (95% CI: 20.0 months–NE), which was significantly different from the result obtained from UR-M PDAC group (median OS 10.0 months; 95% CI: 5.0 months–NE; P = 0.003) but not significantly different from that obtained from BR PDAC group (median OS not reached; 95% CI: 23.0 months–NE; *P* = 0.758) and the UR-LA PDAC group (median OS 22.0 months; 95% CI: 11.0 months–NE; *P* = 0.281) (Figure 7B). In addition, we compared the outcomes between the group for which complete resection was performed and the group for which it was not performed. The median, 1-year, and 3-year OS of the complete resection group were not reached (95% CI: 33.0 months–NE, 86.5, and 64.0%, respectively), indicating significantly different result from that obtained from the “no complete resection” group [median OS 12.0 months; 95% CI: 7.0 months–23.0 months; (*P* ≤ 0.001)]; 56.4%; and 23.9%, respectively (Figure 8). Furthermore, we compared the outcomes between the three groups according to the results of cytological examination of peritoneal washings. The median OS of the Negative cytology group was not reached (95% CI: 18.0 months–NE), which was not significantly different from the results obtained from the Atypical cells detected group (median OS not reached; 95% CI: 5.0 months–NE; *P* = 0.692) and was Positive cytology group (median OS 23.0 months; 95% CI: 4.0 months–NE; *P* = 0.0069) (Figure 9).

## Discussion

SL is a minimally invasive modality for staging PDAC in patients at a high risk of unresectable disease despite CT evidence of resectability and can identify occult metastases in 15–51% of cases (7, 8). The NCCN guidelines suggest that SL should be considered for high-risk patients with PDAC and upfront resectable disease—evidenced by, for example, markedly elevated CA 19–9 levels—and for those with borderline resectable disease before the administration of NAC (9). Rosa et al. proposed that SL should be considered for patients with CT scans suggestive of resectable disease and CA 19–9 levels ≥ 150 U/mL, or a tumor size > 3 cm (10). Karabicak et al. also reported that patients with large pancreatic body-tail tumors and high CA 19–9 levels are at greater risk of developing latent distant organ metastasis or peritoneal metastasis and should undergo SL routinely for accurate diagnosis (11). In the present study, SL revealed distant metastasis in 24 patients (23.5%). Furthermore, of the patients with R PDAC before SL, including those with negative tumor markers, 17.8% were found to have distant metastasis after SL. Multivariate analysis revealed that having a CEA positive status and CA19-9 levels ≥ 150 U/mL were the only factors independently associated with occult metastasis. Clear criteria for conducting SL may be difficult to establish due to variations in reports of patients who should undergo it. However, SL is actively recommended for patients with R PDAC and BR PDAC who are planning to undergo complete resection soon in order to avoid unnecessary surgical exploration, especially those who are CEA positive or with CA19-9 ≥ 150 U/mL.

No morbidity or mortality was reported in the present case series. In a previous meta-analysis that include 3,305 patients who underwent SL, complication rates were minimal (0.5%), and one patient (0.03%) died of myocardial infarction (12). SL is a procedure with a short operative time and a low complication rate. We could able to start chemotherapy approximately 2 weeks after SL, on average. Other reports have also indicated that the median time to start chemotherapy after SL is 10 days (range: 4.0–31.0 days) (5). Furthermore, SL in patients with R PDAC appears to be cost-effective when laparotomy following SL occurs in a subsequent admission (13). These results suggest that SL is safe and that the duration of the chemotherapy delay and the cost-effectiveness are acceptable.

We found that the complete resection rate increased from 80.0 to 94.6% in the patients with R PDAC, from 58.3 to 75.0% in those with BR PDAC, and from 23.8 to 38.5% in those with UR-LA PDAC after SL. With SL, unnecessary surgical exploration can be avoided by detecting nonresectable factors that are undetectable in preoperative imaging, and appropriate treatment can be provided to each patient; however, the improvement in resection rates may partly reflect stage migration rather than true survival benefit.

In Japan, the OS for all PDAC cases between 2001 and 2007 was 14.7 months (14). Previous studies have determined that the OSs is 23.0–46.5 months (15–18) in R PDAC, 14.3–31.5 months in BR PDAC (16, 19, 20), 9.4–33.7 months in UR-LA PDAC (21, 22), and 6.8–11.1 months in UR-M PDAC (23–26). Comparing these findings with the OS after SL in our study, we found comparable results for all resectability statuses. The OS for all cases and resectability statuses tended to be longer than that in existing reports; however, improved outcomes may be driven by better patient selection than by SL itself, and the results of survival analyses before and after SL appear largely driven by stage migration rather than demonstrable treatment benefit. Of the patients with UR-LA PDAC, despite stage migration, no improvement in OS was observed after SL. Further, because the complete resection rate for patients with UR-LA PDAC is low, performing SL before therapy may be less beneficial than for patients with R or BR PDAC.

The pathological distinction between atypical cells and positive cytology may be subtle, and it often depends on the experience of the individual reporting pathologist. There is no consensus yet on how to manage patients with atypical cells. In this study, although no significant difference was observed, the OS of the group with Atypical cells detected was closer to that of the Negative cytology group than to that of the Positive cytology group, and it may be necessary to consider treating them in the same way as the Negative cytology group.

This study has several limitations. First, the sample size might have been relatively small for statistical analysis of many complex factors. Second, the data were collected from a single center, limiting external validity and generalizability. Third, we did not directly compare patients who underwent SL and those who did not, which limits causal inference. Fourth, the exclusion of patients undergoing upfront surgery may introduce selection bias toward healthier patients. Fifth, this study has limited applicability to the elderly population. Sixth, the observation period may be too short for meaningful survival analysis in PDAC. Hence, more studies with data from multiple centers are needed to support our conclusions.

## Conclusion

SL is safe and effective in determining accurate staging, which may allow for more appropriate treatment. Therefore, SL is actively recommended for patients with R PDAC or BR PDAC who are planning to undergo complete resection shortly to avoid unnecessary surgical exploration, especially those CEA positive or with CA19-9 ≥ 150 U/mL.

## Data Availability

The original contributions presented in this study are included in this article/supplementary material, further inquiries can be directed to the corresponding author.
